# Conical-intersection dynamics and ground-state chemistry probed by extreme-ultraviolet time-resolved photoelectron spectroscopy

**DOI:** 10.1038/s41467-018-05292-4

**Published:** 2018-08-08

**Authors:** A. von Conta, A. Tehlar, A. Schletter, Y. Arasaki, K. Takatsuka, H. J. Wörner

**Affiliations:** 1 Laboratory of Physical Chemistry, ETH Zurich, Vladimir-Prelog-Weg 2, CH-8093 Zurich, Switzerland; 2 Fukui Institute for Fundamental Chemistry, Kyoto University, Sakyo-ku Kyoto, 606-8103 Japan

## Abstract

Time-resolved photoelectron spectroscopy (TRPES) is a useful approach to elucidate the coupled electronic-nuclear quantum dynamics underlying chemical processes, but has remained limited by the use of low photon energies. Here, we demonstrate the general advantages of XUV-TRPES through an application to NO_2_, one of the simplest species displaying the complexity of a non-adiabatic photochemical process. The high photon energy enables ionization from the entire geometrical configuration space, giving access to the true dynamics of the system. Specifically, the technique reveals dynamics through a conical intersection, large-amplitude motion and photodissociation in the electronic ground state. XUV-TRPES simultaneously projects the excited-state wave packet onto many final states, offering a multi-dimensional view of the coupled electronic and nuclear dynamics. Our interpretations are supported by ab initio wavepacket calculations on new global potential-energy surfaces. The presented results contribute to establish XUV-TRPES as a powerful technique providing a complete picture of ultrafast chemical dynamics from photoexcitation to the final products.

## Abstract (web summary)

Recent progress in extreme-ultraviolet table-top light sources is enabling advances in ultrafast molecular dynamics. Here the authors demonstrate time-resolved photoelectron spectroscopy with such a source, observing how a molecular wave packet crosses a conical intersection, performs large-amplitude motion and eventually dissociates.

## Introduction

Nearly all photochemical reactions involve a close coupling of electronic and nuclear dynamics. These coupled dynamics determine the dominant pathways for the transfer of charge and energy across molecules^1^. They also determine the outcome of most processes in photochemistry and photobiology^2,3^. Time-resolved photoelectron spectroscopy (TRPES) has been recognized as an outstanding method for probing such dynamics^4–7^ and has led to important insights into, e.g., the photo-stability of nucleobases^8,9^. Other promising emerging techniques include high-harmonic spectroscopy^10,11^, X-ray transient absorption^12–14^, and time-resolved diffraction techniques^15,16^. The uniqueness of TRPES consists in its sensitivity to, both, electronic and nuclear configurations and its accessible theoretical interpretation.

Most experimental work in this field was performed using either ultraviolet-single-photon TRPES (160–400 nm) (e.g.,^17,18^) or multi-photon TRPES (e.g.,^19,20^). The first approach uses probe-photon energies typically smaller than the ionization potential (*I*_p_) of the electronic ground state. The low photon energy limits ionization to a small “observation window” in the configuration space of the excited molecule, from where ionization is energetically possible and allowed by selection rules. This limitation often results in apparent excited-state lifetimes that are shorter than the true lifetimes, as shown in refs. ^21,22^. The low photon energies moreover restrict the variety of accessible final cationic states, which sets limits on the information content of the experimental data. The second approach, multi-photon TRPES, relies on significantly stronger light fields to induce multi-photon transitions to the final cationic states. This approach overcomes the limitations set by the photon energy, but adds complications in terms of weaker selection rules, induced Stark shifts, and intermediate resonances.

**Fig. 1:**
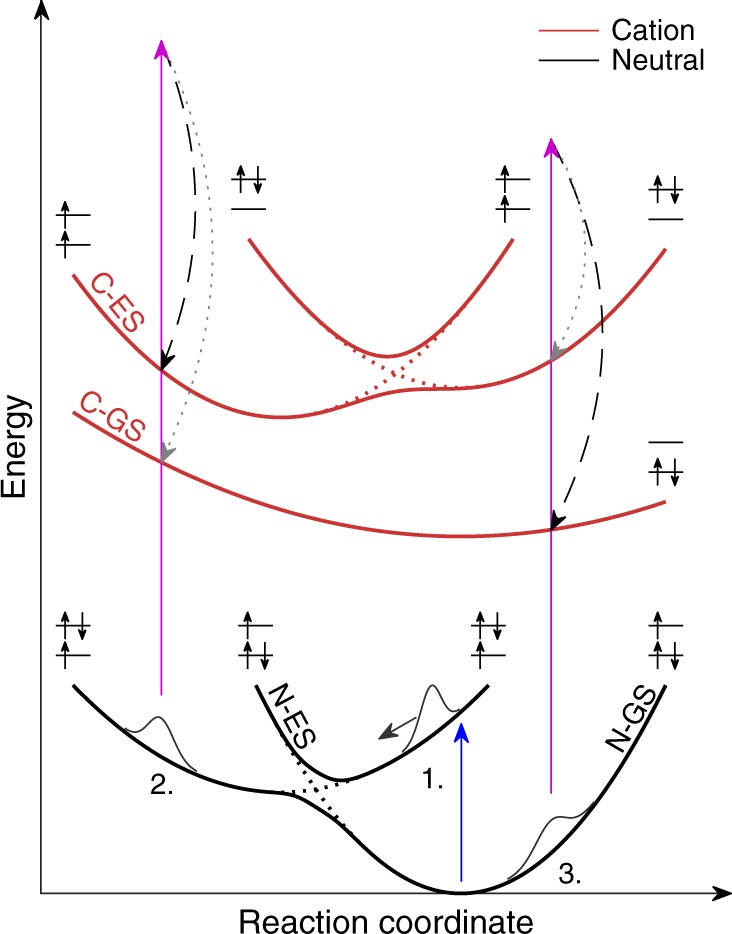
General working principle of extreme-ultraviolet time-resolved photoelectron spectroscopy. The figure shows a set of neutral and cationic potential energy surfaces in adiabatic (solid) and diabatic representations (dashed). At position 1, a WP is generated in the Franck-Condon region of the first exicted state of the neutral (N-ES) with a UV pump photon (blue arrow). As the excited-state WP moves along the potential surface, the vertical *I*_p_ to the cationic groundstate (C-GS) and the first cationic excited state (C-ES), never exceeds the XUV photon energy (violet arrow). During the propagation of the WP, the electronic character changes (depicted as the leading electronic configurations), therefore changing the electronic overlap between neutral and cationic states. At position 2, the transition to the C-GS is Koopmans forbidden (dotted gray arrow) while the transition to the C-ES is allowed (dashed black arrow). Upon relaxation of the WP to the ground-state of the neutral (N-GS), the effect is inverted (position 3)

In this article, we outline the general working principles of XUV-TRPES for polyatomic molecules. Figure 1 illustrates the general concept underlying this technique. A nuclear wave packet (WP) is generated on an electronically excited state of the neutral molecule. This WP then moves along the potential-energy surface, accessing various regions of nuclear configuration space. When the WP reaches a region of strong non-adiabatic coupling, the electronic character of the WP changes, corresponding to population transfer between the two interacting electronic states. Using an XUV pulse, ionization from the neutral to the cationic states is possible at all configurations, including the neutral ground state. The ionization probability is defined by the magnitudes of the photoionization matrix elements, which are usually only large when the leading configurations of the initial and final states differ through the removal of a single electron (Koopmans’ correlation). The high photon energy therefore removes the limitations of UV-TRPES and accesses multiple final states that offer complementary information on the evolving electronic and nuclear structures of the photoexcited WP. XUV-TRPES gives immediate access to the induced population transfer, by monitoring the initial depletion of the ground-state signal, as well as the dissociation dynamics, by the emergence of the photoelectron spectra of the final photoproducts. The complete underlying molecular dynamics can be inferred through comparison with a high-level theoretical model.

Previous work on XUV-TRPES has concentrated on the photodissociation dynamics of Br_2_^23–26^ and two-photon-excited molecules^27,28^. Very recently, the extension to single-photon excitation has been demonstrated^29,30^, but spectral overlap between the photoelectron bands of excited and unexcited molecules has prevented the full potential of XUV-TRPES from being exploited.

In our work, we show that the ability of XUV-TRPES to project the photoexcited WPs onto multiple final cationic states gives access to the complete dynamics of a molecular WP from photoexcitation, over conical-intersection dynamics to large-amplitude motion and dissociation in the electronic ground state. The technique provides a minimally biased picture of the dynamics of a photo-excited molecule, simultaneously characterizing the evolution of the electronic and nuclear configurations. Our detailed analysis identifies individual photoelectron bands that reveal time-dependent electronic configuration, wave-packet motion through a conical intersection (CI) and large-amplitude nuclear dynamics in the electronic ground state. These interpretations are supported by quantum-mechanical wave-packet calculations on three-dimensional potential energy surfaces calculated at a high level of ab initio theory. The advantages of XUV-TRPES come at the cost of challenges that we overcome through a number of innovations. The high photon energies unavoidably lead to overlap between the photoelectron spectra of the excited and unexcited molecules. Implementing spectral subtraction on a single-shot basis, we retrieve high-quality difference spectra. The depletion of the ground-state population through photoexcitation leads to overlapping gain and loss contributions in these difference spectra. We introduce a general method for removing the depletion contributions, which simultaneously provides the complete broadband photoelectron spectra of the excited molecules and an experimental measure of the excitation fraction. Finally, we identify the laser-assisted photoelectric effect (LAPE) as a significant contribution to the time-resolved spectra and introduce a general technique for removing its contributions.

## Results

### The ultrafast dynamics of NO_2_

The system chosen to demonstrate this approach is NO_2_. Several recent studies were dedicated to the non-adiabatic dynamics in NO_2_ using multi-photon TRPES^19,31–34^, time-resolved ion spectroscopy^35^ and high-harmonic spectroscopy^11^. The non-adiabatic dynamics of NO_2_ have also been studied theoretically^36–39^. For a recent review on NO_2_ see ref. ^40^. The strong non-adiabatic coupling between the electronic ground state (GS) and the first excited state allows us to follow a WP, from excitation, to electronic relaxation through a CI and the ensuing dissociation on the ground-state surface. This extends TRPES to ultrafast photo-induced chemistry on the electronic ground state, naturally inaccessible to UV-TRPES.

**Fig. 2:**
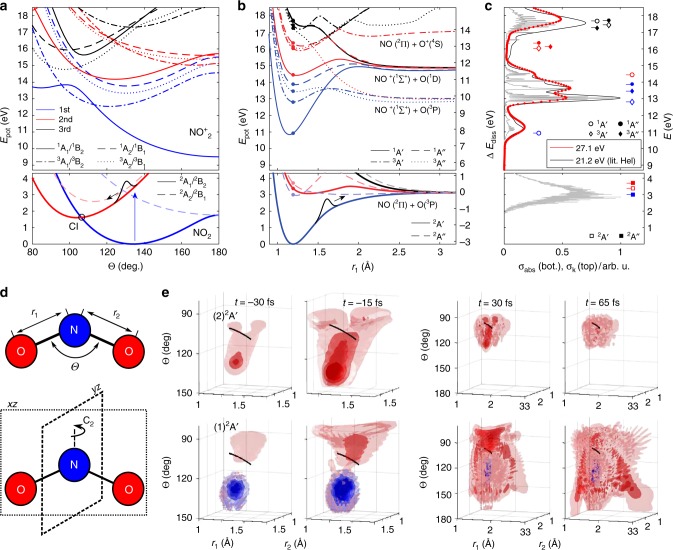
Electronic structure and dynamics of NO_2_. **a** Adiabatic potential energy curves as a function of the bond angle Θ. The calculations are performed for equal bond lengths $r_1 = r_2 = r_{{\mathrm{eq}}}^{{\mathrm{exp}}} = 1.193$ Å in *C*_2v_ symmetry. The colors blue, red and black label the lowest, second lowest and third lowest potential-energy surfaces of each symmetry type defined by the nature of the line (full, dashed, dash-dotted, dotted), as indicated in the legend. **b** Same curves as function of *r*_1_, calculated in *C*_s_ symmetry, with *r*_2_ and Θ set to the experimental equilibrium values. The energy axis on the right-hand side of this panel is given relative to the dissociation threshold. **c** Comparison between a high-resolution photoelectron spectrum (gray)^59^ convoluted with the experimental energy resolution (black), the experimental photoelectron spectrum obtained in this work (red) and the calculated position of the individual photoelectron bands according to the curves from **b**. In the same panel (bottom part) a UV-absorption spectrum^60^ (gray curve) is compared to **b** (square markers, colored according to **b**). **d** The coordinate conventions used throughout this article. **e** Time slices from the WP calculation described in the text and Supplementary Note 1; the top figures show the WP density on the adiabatic excited state and the lower row of figures shows the population change of the adiabatic ground-state. The isodensity coloration is blue for a depletion of initial population and red for a population gain. The black line depicts the seam of CIs

In this work, NO_2_ is excited by an ultrashort UV pulse, centered around 3.11 eV (398 nm) with a full width at half maximum (FWHM) of 50 meV (≈6 nm) and a peak intensity of (1.2–2) × 10^11^ W/cm^2^. The induced dynamics are particularly interesting because of their complex nature, caused by the CI between the two participating states. Figure 2 shows cuts through our new global potential energy surfaces of NO_2_ and ${\mathrm{NO}}_2^ +$ along the bond-angle (a) and along one N–O bond-distance coordinate (b). The induced dynamics are probed by an ultrashort XUV pulse centered at 27.1 eV from a time-preserving monochromator^41^. The relative polarization of UV and XUV pulses was parallel in all experiments. Figure 2c shows the high-resolution photoelectron spectrum of NO_2_ from the literature, superimposed with the photoelectron spectrum obtained in this work. The lower part of the panel shows the photoabsorption spectrum of NO_2_. The complexity of the energy-level structure in this energy region is so high that a complete understanding of the high-resolution spectra is still to be achieved.

Following excitation to the Franck–Condon (FC) region of the (2)^2^A′ surface, the WP undergoes electronic relaxation to the (1)^2^A′ ground state through the CI. Here and in what follows, we use symmetry labels of the C_s_ point group because this point group is appropriate for all geometries of NO_2_. We additionally provide the symmetries in the C_2*v*_ group in square brackets where required.

The results of our three-dimensional quantum wave-packet calculations on our new potential-energy surfaces are displayed in Fig. 2e. For details on these calculations, see 'Methods' and Supplementary Note 1. These potential-energy surfaces feature, to the best of our knowledge, the highest accuracy and widest range of nuclear coordinates sampled to date. The latter represents an essential improvement, as the dissociation of NO_2_ which is discussed below, could previously not be accurately described. Figure 2e shows temporal snapshots of the calculated WP motion for a 40 fs pump pulse centered at 400 nm. The time convention is that 0 fs corresponds to the maximum of the pump-pulse envelope. The lower row shows the part of the WP residing in the lower adiabatic state and the upper panels show the WP in the adiabatic excited state. The first time slice is for *t* = −30 fs, at a time where the pump pulse starts interacting with NO_2_. The depletion of the ground-state wavefunction is visible as a concentrated blue sphere in the lower panel, while the upper panel shows the population appearing at the same position in the FC region. The population transferred to the excited state starts moving towards the seam of CIs, displayed as a black line. Parts of the nuclear WP have scattered, visible as two lobes next to the CI and population is transferred to the lower adiabatic state appearing as population above the CI. The second snapshot at −15 fs illustrates the reversal of the WP motion in the angular coordinate. At this time, the leading edge of the WP, located on the upper surface, has turned around, while the WP on the ground-state surface is still running up to its turning point. The pulse envelope has not reached its maximum yet and population is continuously generated in the FC region. In the third set of panels, the density is shown for *t* = 30 fs, at the end of the interaction window. The motion from the FC region to the CI is still clearly visible on the upper surface, however, the WP has also spread out in a confined region of the configuration space. On the lower adiabatic state, the WP is spreading out towards large angles and long bond distances. Sixty five-fs after the peak of the envelope, the population transfer ceases and there is a diffuse WP left in the upper state. On the ground-state surface, two dissociation pathways become visible. The two lobes at large *r*_1_(*r*_2_) for small *r*_2_(*r*_1_), moving towards large angles, represent the dissociation of one of the two oxygen atoms, while the other N–O distance remains close to the equilibrium geometry of NO. We note that the WP density remains symmetric with respect to the permutation of *r*_1_ with *r*_2_ as required.

According to the topology of our potential energy surfaces, dissociation of NO_2_ at the excitation energies used in our experiment happens exclusively via the (1)^2^A′ state. The NO(^2^Π_Ω_) + O(^3^P_*J*_) dissociation threshold is located at an energy of 3.116 eV for Ω = 1/2 and *J* = 2^42^. At the employed excitation energies, photoabsorption is entirely dominated by the (2)^2^A′ [(1)^2^B_2_] state, such that the induced WP is therefore confined to the (1)^2^A′ and (2)^2^A′ surfaces.

### Depletion-corrected excited-state photoelectron spectra and their time dependence

**Fig. 3:**
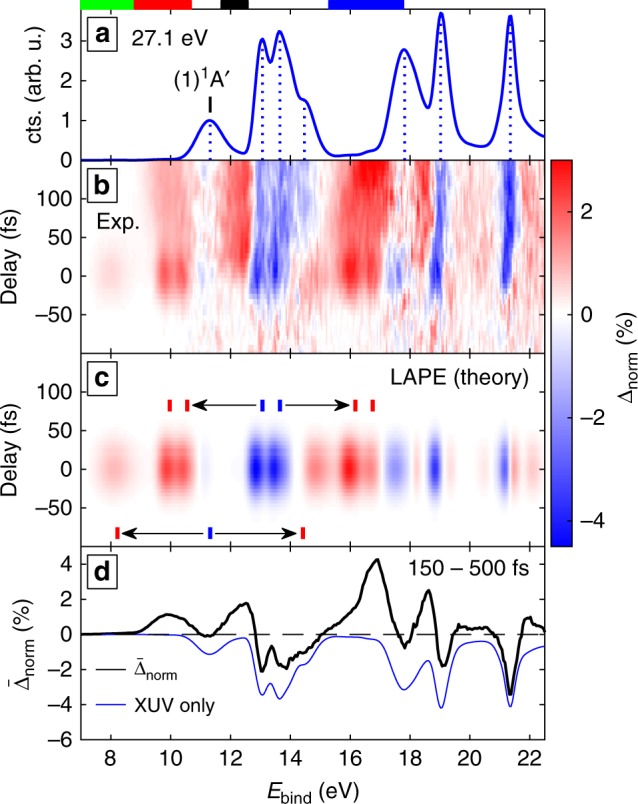
Description of the data analysis and laser-assisted photoelectric effect (LAPE). **a** Experimental photoelectron spectrum of unexcited NO_2_ obtained with an XUV photon energy of 27.1 eV. The dashed blue lines indicate the peak position of each band. The color bars label the energy domains that are analyzed further in Figs. 4 and 5 (**b**) Experimental difference spectrum showing the excited-state photoelectron bands in red (gain) and depletion of XUV-only peaks (blue). The colormap is given in percent of the (1)^1^A′ peak in the XUV-only spectrum. The time convention is that for negative times the XUV pulse comes first. **c** Calculated LAPE spectrum (see text). The arrows indicate which photoelectron band (blue dashes) is shifted to which final position by the energy of one UV photon (3.1 eV) (red dashes). **d** Difference spectrum averaged over the delay range from 150 to 500 fs. An XUV-only spectrum is shown in the same plot in order to highlight depletion dominated regions

Figure 3 shows the experimental results for short pump-probe delays. Because of the high photon energies used in XUV-TRPES and the small excitation fractions, limited by the onset of multi-photon ionization by the pump pulse, the photoelectron spectra are always dominated by the contribution of unexcited molecules, shown in Fig. 3a. Therefore, we show throughout this work normalized-difference spectra,

1 $${\mathrm{\Delta }}_{{\mathrm{norm}}} = \frac{{\left[{\mathrm XUV} + {\mathrm UV} \right] - \left[{\mathrm{XUV}} \right]}} {{\left[{\mathrm{XUV}, (1)^1{\mathrm A}'} \right]}},$$

normalized to the maximum of the (1)^1^A' band, where [XUV + UV] and [XUV] represent the photoelectron spectra obtained in the presence of the pump and probe pulses, or the probe pulse only, respectively. Figure 3b shows Δ_norm_ as a function of the pump-probe delay. Positive signals (red) correspond to photoelectrons from photoexcited molecules, whereas negative signals (blue) are dominated by the depletion of the population of unexcited molecules in the vibronic ground state. The corresponding spectra [XUV] and [XUV + UV] can be found in the Supplementary Note 2 and Supplementary Fig. 4 together with a detailed description of the applied data processing.

We first focus on the region of zero delay, corresponding to temporal overlap of the pump and probe pulses. The spectral signatures observed in this region can almost perfectly be explained by the LAPE, (see Fig. 3c). This effect has previously been observed in photoemission from atoms^43,44^, but has not been discussed in the case of isolated molecules. Therefore, we have developed a simple model (see 'Methods' and Supplementary Note 3) to calculate LAPE spectra using strong-field perturbation theory and utilizing experimental XUV photoelectron spectra as input. The result of these calculations using the experimental conditions is shown in Fig. 3c.

The LAPE contributions are negative at positions of the [XUV] photoelectron bands and positive at the corresponding positions offset by the energy of one UV photon. The reason why the gain features (red) are most intense at low binding energies is that the LAPE effect depends on the final energy of the created photoelectron. For high binding energies (small photoelectron kinetic energies), the effect is suppressed.

**Fig. 4:**
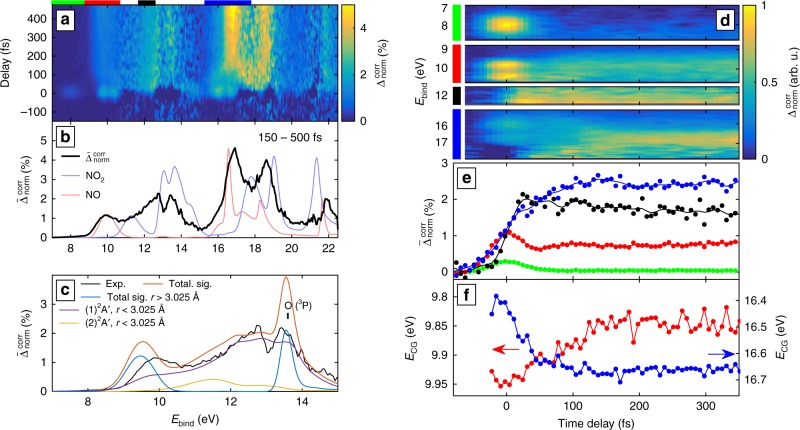
Overview of the depletion-corrected excited-state photoelectron spectrum and its time dependence. **a** Dataset from Fig. 3b) after depletion correction (see text). The excitation was determined to be 1.3%. **b** Mean difference spectrum outside of the temporal overlap (cf. Fig. 3d). XUV only spectra of both NO_2_ and NO are overlayed to indicate the origin of individual contributions. The NO spectra were acquired with a different magnetic-bottle spectrometer^61^. **c** Same data with the region between 7 and 15 eV magnified and superimposed calculated spectra. The binding energy range matches the energies where the cationic states included in our model, suffice to describe the system. The corresponding energy domains are labeled by bars of the same colors in **a**. **d** The four selected bands described in the text, each renormalized to a peak amplitude of 1. **e** Mean variation of each band as a function of time delay and **f** The center of gravity calculated for two selected bands. The color code of the bands in **d**–**f** is identical

Both effects, LAPE as well as the overlapping depletion and gain features (see Fig. 3d) obfuscate the interpretation. We have therefore developed another new approach, which allows for the cancellation of the depletion features. Our method consists in constructing a normalized-difference spectrum by integration over large positive delays (Fig. 3d) and adding to it the original [XUV] spectrum weighted by the excitation fraction. Ensuring the continuity of the obtained spectrum as a function of binding energy results in a unique determination of the excitation fraction at asymptotically large pump-probe delays. The result of this procedure (detailed in the Supplementary Note 4) are depletion-corrected normalized-difference spectra ${\mathrm{\Delta }}_{{\mathrm{norm}}}^{{\mathrm{corr}}}$. An example using the data from Fig. 3 is displayed in Fig. 4b.

This approach has three immediate benefits. First, it provides the complete photoelectron spectrum of the photoexcited molecules, over an extremely large bandwidth (>6 eV in this case), devoid of depletion effects. Second, it offers an experimental measure of the excitation fraction, which is usually not easily accessible in TRPES. Finally, knowing or assuming the envelope function of the pump pulse, the depletion can even be compensated at each time step during temporal overlap of pump and probe pulses by adding the [XUV] spectrum weighted with the integral of the envelope function of the pump pulse (see 'Methods'). The process as a whole is shown in detail in the Supplementary Note 4.

Figure 4a shows a depletion-corrected difference spectrum ${\mathrm{\Delta }}_{{\mathrm{norm}}}^{{\mathrm{corr}}}$ as a function of the pump-probe delay. Figure 4b shows the temporal average $\overline {\mathrm{\Delta }} _{{\mathrm{norm}}}^{{\mathrm{corr}}}$ of ${\mathrm{\Delta }}_{{\mathrm{norm}}}^{{\mathrm{corr}}}$ over the delay range from 150 to 500 fs. We have verified that the photoelectron spectra remain unchanged over this time interval, such that its choice does not affect the depletion correction. Since the spectral features visible in Fig. 4a do not change significantly for delays longer than 150 fs, and our calculations indicate that most of the population transfer is completed by this time (Supplementary Fig. 1), the black spectrum displayed in Fig. 4b represents the photoelectron spectrum of photoexcited NO_2_ molecules. This represents the first observation of the broadband photoelectron spectrum of an excited molecule. This spectrum can be compared to the overlayed photoelectron spectra of NO_2_ and NO in their lowest respective vibronic states, shown as blue and red lines in Fig. 4b, respectively. The dominant features of both spectra coincide with regions of high signal strength in the spectrum of photoexcited NO_2_ molecules. The latter is however substantially broader than the spectra of both unexcited molecules. This property is a direct consequence of the delocalization of the photoexcited WP in nuclear-coordinate space, as predicted by our wave-packet calculations (Fig. 2e). Figure 4c shows a direct comparison of this broadband photoelectron spectrum with our calculations that extend to binding energies of ~15 eV. The good overall agreement shows the high accuracy of our potential energy surfaces and validates our calculations of photoelectron spectra, even for photoexcited molecules. The overestimation of the signal associated with dissociation products (e.g., O(^3^P)) results from a slight underestimation (by ~0.09 eV) of the dissociation threshold in our calculations.

### Observation of CI dynamics and large-amplitude motion

We now discuss the femtosecond dynamics of NO_2_ revealed by our measurements. We focus on three time-dependent photoelectron bands, which are shown in detail in Fig. 4d–f and labeled by colored bars above Figs. 3a, 4a. Band 1 (green, 7 to 8.8 eV) contains a single cross-correlation-like feature around zero delay. Band 2 (red, 8.8 to 10.7 eV) contains two similar structures, as well as a persisting feature outside the pulse overlap. Band 3 (black, 11.7 to 12.6 eV) shows a step-like signal appearance which appears shifted from zero delay. The fourth displayed band (blue, 15.3 to 17.8 eV) covers the region of the dominant NO band, showing its gradual appearance. However, this band lies outside the binding energies covered by our cationic surfaces and can therefore not be compared to our calculations.

The following interpretation of our results and our detailed calculations of time-dependent photoelectron spectra rely on the reflection principle, which has previously been validated in TRPES^45^. Briefly, the reflection principle gives an expression for the time-dependent ionization cross section *σ*_E_(*E*_bind_,*t*) depending only on the nuclear density in the neutral electronic states $\left| {\Psi _i\left( {\vec R,t} \right)} \right|^2$, the vertical $I_{\mathrm{p}}^{if}\left( {\vec R} \right) = \varepsilon _f\left( {\vec R} \right) - \varepsilon _i\left( {\vec R} \right)$ between neutral (*i*) and cationic states (*f*), and the electric-dipole photoionization matrix element between initial and final electronic states, $\mu _{if}^e\left( {\vec R} \right)$,

2 $$\sigma _{\mathrm{E}}\left( {E_{{\mathrm{bind}}},t} \right) \propto \mathop {\sum}\limits_{i,f} {\int} \left| {\mu _{if}^e\left( {\vec R} \right)} \right|^2\,\delta \left( {E_{{\mathrm{bind}}} - I_{\mathrm{p}}^{if}\left( {\vec R} \right)} \right)\;\left| {{\mathrm{\Psi }}_i\left( {\vec R,t} \right)} \right|^2d\vec R,$$

with further details given in 'Methods' and Supplementary Note 5.

**Fig. 5:**
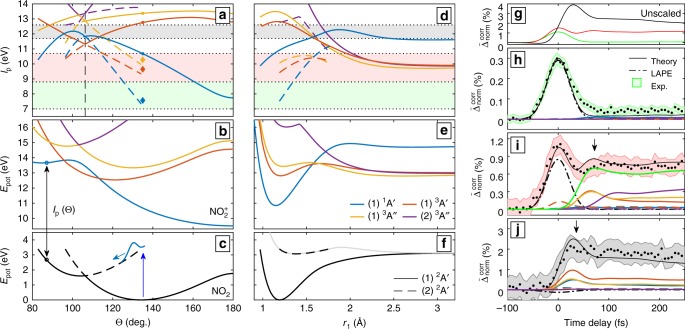
Quantitative interpretation of the time dependence of three selected photoelectron bands. **a**–**c** Influence of the bond angle Θ on the local *I*_p_, where **b**, **c** display the potential energy curves of the neutral states and the cationic states, respectively, and **a** shows the vertical *I*_p_ highlighting the selected photoelectron bands (see Fig. 4d). The color code corresponds to **b** distinguishing transitions from the lower adiabatic state (solid lines) and transitions from the upper adiabatic state (dashed lines). Diamond markers indicate LAPE contributions. The vertical dashed black line in **a** indicates the CI. **d**–**f** show the dependence of the bond distance *r*_1_. The gray curve in **f** indicates the complete upper adiabatic state, while the dashed black line emphasizes the accessible part of this state (see text). **g** Calculated mean band variation (see Fig. 4e). **h**–**j** Comparison of experimental data with the rescaled calculated traces. The shaded regions represent the standard deviation of the mean, obtained from 20 consecutive measurements. Contributions from the individual states are displayed using the color code from **a**, **d**. The additional green line in **i** shows the contribution of the three triplet states, i.e., from large amplitude motion, its peak at 72 fs is highlighted by an arrow. The arrow in **j** indicates the peak contribution of the signal associated with the CI region of the nuclear configuration space at 36 fs

A given molecular geometry $\vec R$ can therefore only contribute to a photoelectron band if the local $I_{\mathrm{p}}^{if}\left( {\vec R} \right)$ lies within the edges of the band for a given combination of initial and final states. This provides an intuitive interpretation of the WP motion by looking at the variation of $I_{\mathrm{p}}^{if}\left( {\vec R} \right)$ as a function of the nuclear coordinates. This dependence is illustrated in Fig. 5a, d where bands 1 to 3 are indicated by the colored areas, matching the color code established in Fig. 4. The lower (upper) adiabatic states of the neutral molecule and the corresponding vertical ionization potentials are represented by full (dahsed) lines. Figure 4h-j compares the experimental measurements with the results of our wave-packet calculations. Since the individual line strengths of the calculated spectra deviate slightly from the experiment, the calculated traces were rescaled when compared to the experiment (cf. Figs. 4e, 5g).

Band 1 (Fig. 5h) is dominated by a LAPE contribution at zero delay. This contribution is identified in Fig. 5a by a blue diamond, placed by one UV-photon energy (3.11 eV) below the blue curve, in the FC region of the lower adiabatic state. In contrast to this dominant LAPE feature, the expected contribution from ionizing the upper adiabatic state to the (1)^1^A′ state is negligible. The nearly complete suppression of this signal demonstrates the sensitivity of XUV-TRPES to the electronic character of the excited-state WP, as illustrated in Fig. 1. In the present case, this is a consequence of the fact that the leading configuration of the (1)^1^A′ state of ${\mathrm{NO}}_2^ +$ [(…)(4b_2_)^2^(1a_2_)^2^] at the GS equilibrium geometry differs by more than one electron from that of the (2)^2^A′ state of NO_2_ [(…)(4b_2_)^1^(1a_2_)^2^(6a_1_)^2^]. The small signal contributions outside of the temporal overlap of pump and probe pulses originate from quasi-linear molecules in the lower adiabatic state, which has the correct electronic configuration [(…)(4b_2_)^2^(1a_2_)^2^(6a_1_)^1^] at large bond angles to be efficiently ionized to the (1)^1^A′ state. The slow rise of this signal with the delay therefore reflects the arrival of wave-packet density in the quasi-linear region of the lower adiabatic state. This interpretation follows from the fact that the full blue line in Fig. 5a only overlaps with the green band for *θ* > 160°. The dashed blue lines also overlap with the green band, but the corresponding contributions from the upper adiabatic state are negligible because of the small electronic overlap discussed above. These complementary observations reflect the pronounced sensitivity of XUV-TRPES to the electronic configurations of the neutral and ionic states on one hand, and to the nuclear geometries on the other.

Band 2 (Fig. 5i) contains two LAPE contributions from the lowest two triplet states of ${\mathrm{NO}}_2^ +$ that dominate the zero-delay range. This explains the maximum of band 2 at zero delay. The signal outside time zero is almost exclusively contributed by ionization from the large-bond-length areas of the lower adiabatic state (*r*_1_ > 2 Å) to the same two triplet states and, additionally, the (2)^3^A″ state of ${\mathrm{NO}}_2^ +$ (see Fig. 5d). As a consequence, the observed second maximum in band 2 (at a delay of 72 fs in the calculation, arrow in Fig. 5i) is a direct signature of the large-amplitude motion of the WP as it accesses the configuration space corresponding to large bond lengths. The center of gravity of the experimental photoelectron band shifts by about 100 meV towards lower binding energies (red curve in Fig. 4f). This observation is also consistent with the WP motion towards long bond lengths. As shown in Fig. 5d, the vertical *I*_p_ of the contributing cationic states decreases towards large *r*, supporting the observed trend. The calculated and measured band strengths agree well with each other and thereby support the intuitive explanation given above. The projected densities of the WP in *r* and Θ, supporting this interpretation, can be found in the Supplementary Fig. 1.

Band 3 (Fig. 5j) does not contain any observable LAPE contributions and is, at the same time, free of contributions from large-amplitude motion. Its main contributions originate from the region of configuration space corresponding to a bond angle between 90 and 110° and are dominated by transitions from the lower adiabatic state. This means that the delay between the onset of this band and *t* = 0 is directly related to the WP accessing the CI. As the WP spreads on the ground-state surface the relative population of this region remains constant at later times, except for the dissociating fraction. Indeed, band 3 peaks around 36 fs, significantly before band 2. The time scale agrees well with the onset of re-population of the lower adiabatic state, shown in the Supplementary Fig. 1.

### Identification of photodissociation and ground-state dynamics

**Fig. 6:**
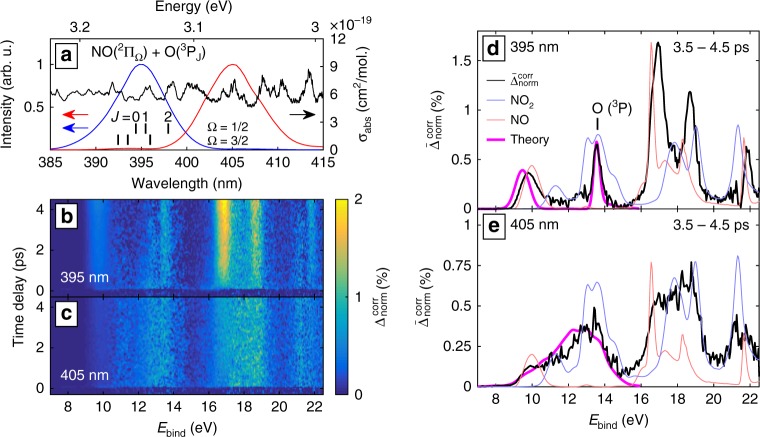
Photoelectron spectra of dissociated and highly-excited, but still bound NO_2_ molecules. **a** The wavelength-dependent absorption spectrum of NO_2_ (black)^60^ with the individual dissociation thresholds for vibrationally cold NO_2_^62, 63^, together with two excitation spectra—one dominantly above (blue) and one dominantly below (red) the dissociation threshold. **b**, **c** Depletion compensated relative difference spectra for those two excitation spectra. The excitation fraction in both data sets (**b**, **c**) is about 0.9%. **d**, **e** Mean depletion -compensated relative difference for both spectra integrated over the delay range of 3.5 to 4.5 ps. In both cases the XUV-only spectra of NO_2_ and NO are overlayed (see text). The NO spectra were acquired with a different magnetic-bottle spectrometer^61^. The photoelectron spectra predicted by our wave-packet calculations are shown as well

Another strength of XUV-TRPES lies in its ability to follow the photo-induced dynamics up to arbitrarily long delays, allowing for the observation of, both, the final photoproducts and the dynamics of vibrationally hot molecules in the electronic ground state. In our experiment, we demonstrate this opportunity by tuning the spectrum of our excitation pulse above or below the dissociation threshold. Figure 6a shows the two employed pump spectra, one centered around 395 nm (3.13 eV) and one at 405 nm (3.06 eV).

In the dissociating case (pump spectrum centered at 3.13 eV, Fig. 6b), the gradual appearance of relatively sharp photoelectron bands can be observed. Each of these bands can be assigned to one of the photofragments, NO(^2^Π_Ω_) and O(^3^P), as shown in Fig. 6d.

In the non-dissociating case (pump spectrum centered at 3.06 eV, Fig. 6c), one observes the appearance of a broad, comparably less structured photoelectron spectrum that does not show any pronounced time dependence. The corresponding broad-band photoelectron spectrum of the photoexcited molecules $\overline {\mathrm{\Delta }} _{{\mathrm{norm}}}^{{\mathrm{corr}}}$ (Fig. 6e) shows remarkable differences as compared to the spectrum in Fig. 6d. The spectrum of the non-dissociated molecules appears to be composed of a superposition of a somewhat broadened version of the NO and NO_2_ photoelectron spectra. This is a clear signature that the photoexcited WP spans very large regions of configuration space at long delays. In particular, the WP covers regions close to the ground-state equilibrium geometry, resulting in features that resemble the spectrum of unexcited NO_2_, and regions with one quasi-dissociated oxygen atom, yielding NO-like features in the photoelectron spectrum.

These observations are the first experimental evidence in photoelectron spectroscopy of the population of loosely bound states in molecular photodissociation. The geometry of these states correspond to an O atom at a large distance from the NO moiety, that itself has a geometry close to the equilibrium geometry of isolated NO. This class of states has been proposed as an explanation for the considerable increase in the density of states of NO_2_ between 0 and 100 cm^−1^ below its dissociation threshold^46^. These loosely-bound states are closely related to the roaming-atom mechanism in photodissociation^47^. The ability of XUV-TRPES to identify such states suggests this technique as a potentially useful approach to elucidate the roaming-atom mechanism from a new perspective.

Finally, we briefly compare the results of the present study with previous investigations of the dynamics of NO_2_ excited at 400 nm. Together with previous studies using time-resolved high-harmonic spectroscopy (TRHHS)^11^, the present results represent the first clear observation of the NO_2_ dynamics following single-photon excitation with femtosecond time resolution. The hallmark of single-photon excitation in both cases is the picosecond photodissociation dynamics, and its suppression for pump-photon energies lying below the dissociation threshold. These dynamics also agree with earlier studies using laser-induced fluorescence (see e.g.,^48,49^). The femtosecond dynamics observed by high-harmonic spectroscopy^11^ showed a time-dependent oscillation of the diffracted signal featuring 1-2 oscillations with a period of ~100 fs. Whereas band 2 (Fig. 5i) also displays two maxima separated by ~100 fs (in the experimental data), there is no direct equivalence between the measured signals, for at least two reasons. First, the sensitivity of TRHHS to specific regions of configuration space is influenced by the coordinate dependence of the strong-field ionization rate, in addition to that of the photorecombination matrix elements, the latter being the complex conjugates of the photoionization matrix elements governing the sensitivity of XUV-TRPES. Second, XUV-TRPES resolves the final state of the cation in contrast to TRHHS, which measures the coherent sum of the high-harmonic emission from multiple channels. In spite of these additional complications, our improved potential energy surfaces and detailed WP calculations may also turn out to be helpful in reaching a quantitative understanding of the TRHHS results.

## Discussion

In this work we have demonstrated that XUV-TRPES can provide a complete picture of ultrafast molecular WP dynamics. It was shown that both the excited-state fraction and the dissociation dynamics are immediately accessible. We have also recovered signatures of the CI dynamics and the large-amplitude motion of vibrationally hot ground-state molecules. The overall behavior, as well as the recovered time scales agree well with the observables extracted from the 3D quantum-WP calculations on new, global potential-energy surfaces.

As a consequence of the projection on multiple final states, XUV-TRPES provides a multi-dimensional view of the coupled electronic and nuclear dynamics. The technique moreover removes the limitations that the narrow observation window imposes on UV-TRPES. In combination with single-photon excitation, broadband photoelectron spectra of photoexcited molecules can be extracted and followed over the entire dynamical evolution from photoexcitation, over conical-intersection dynamics to the final photoproducts. These time-dependent photoelectron spectra reveal the true dynamics of the photoexcited molecules with minimal bias from the probing technique. In future experiments, additional information can be obtained by recording time- and final-state resolved photoelectron angular distributions^50^.

The experimental and theoretical methodology developed here can be directly applied to other systems. To the best of our knowledge, this is also the first study showing LAPE from isolated molecules together with a semi-quantitative model. Our work opens new avenues towards the understanding of more complex molecular systems and their dynamics, such as the non-adiabatic photochemical dynamics of molecules in all phases of matter.

*Note added at proof*: We note the recent publication of ref. ^51^, reporting a beautiful XUV-TRPES study of CS_2_.

## Methods

### Description of the experimental setup

A HHG-based XUV monochromator provides 35 fs XUV pulses which are interferometrically recombined with 44 fs UV pulses in an angle-integrating magnetic-bottle spectrometer, providing an energy resolution of better than 200 meV over the displayed energy region. The experimental energy resolution of 300 meV is defined by the energy bandwidth of the XUV. The delay between the two pulses is set by changing the optical path length of the UV pulse using a motorized translation stage. NO_2_ is delivered into the interaction region of the spectrometer by means of a needle-type leak valve with active pressure stabilization. In order to limit the formation of N_2_O_4_ the leak valve was heated to 90 °C. At this temperature, less than 10% of the total pressure in the interaction region is due to N_2_O_4_. No signatures of N_2_O_4_ were observed in the photoelectron spectra, as can be judged by comparing Fig. 3a to ref. ^52^. For details see the Supplementary Note 2.

### Calculation of potential energy surfaces

The energetically lowest two ^2^A′ states of NO_2_ and the two energetically lowest states of ^1^A′, ^1^A″, ^3^A′, and ^3^A″ symmetry of ${\mathrm{NO}}_2^ +$ were calculated with the multireference internally contracted configuration interaction (MRCI) program in the molpro suite^53^ using 3 core, 2 closed and 10 active orbitals with two-state averaging and the aug-cc-pVQZ basis set. The neutral states were diabatized with the phenomenological method described in ref. ^54^.

### Calculation of the vibronic wavepackets

The excitation of the vibronic WP and propagation on the coupled neutral surfaces were performed with the method described in ref. ^37^. In short, we determined the vibrational ground state of the system and, assuming aligned molecules, explicitly included the dipole-coupling of the states due to the pump pulse. The wave functions were propagated with a split-step-operator method, transforming between the adiabatic frame to propagate on the vibronic potentials and the diabatic frame to propagate with the nuclear kinetic-energy operators. The Fourier-limited pump pulse had a peak intensity of 2 × 10^11^W/cm^2^ and a FWHM of the Gaussian intensity envelope of 40fs, leading to an excitation fraction of 3.6%. The results of the calculation are shown in the Supplementary Note 1.

### Calculations of the photoelectron spectra

We use the semi-classical reflection principle^55,56^, to calculate the time-dependent photoelectron spectra. In this work, the quantity $\mu _{if}^e\left( {\vec R} \right)$ is approximated as $\mu _{if}^{red}\left( {\vec R} \right)$, the overlap between the ionized neutral electronic wavefunction and the individual cationic states, disregarding the continuum wave function of the liberated electron. The first two cationic states of *A*′ and *A*′′ symmetry for both singlet and triplet spin states are taken into account (see Fig. 2a, b). In Fig. 2c, the vertical $I_{\mathrm{p}}^{if}$ at the ground-state equilibrium coordinates is compared to an experimental photoelectron spectrum, the calculated binding energies are indicated by colored markers. A calculated static photoelectron spectrum using Eq. 2 and the measured photoelectron spectra are shown in Supplementary Fig. 9. The time and energy resolutions of the experiment were introduced by convolving the calculated signal with a corresponding Gaussian with 0.35eV FWHM in energy and 35fs FWHM in time. Population which was lost during the propagation due to absorption at the edge of the grid (*r*_1_ or *r*_2_ ≥ 3.0263 Å) was assumed to dissociate and associated with an average photoelectron spectrum. Details can be found in the Supplementary Note 5.

### LAPE calculations

The LAPE photoelectron spectra were calculated using strong-field perturbation theory^57^^,^^58^. The formalism was adapted to use an experimental photoelectron spectrum as input, treating each component of the spectrum as an individual non-interacting photoelectron continuum. For further information see the Supplementary Note 3.

### Data availabilty

The data that support the findings of this study are available from the corresponding author upon reasonable request.

## Electronic supplementary material

Supplementary Information
