# Supplementary material for: Conical-intersection dynamics and ground-state chemistry probed by extreme-ultraviolet time-resolved photoelectron spectroscopy
Source: Nat Commun. 2018 Aug 8;9:3162. doi: 10.1038/s41467-018-05292-4 (PMC6082858; doi:10.1038/s41467-018-05292-4)
Supplement: Supplementary file 1 — Supplementary Information [file 41467_2018_5292_MOESM1_ESM.pdf]

Conical-intersection dynamics and ground-state chemistry probed by extreme-ultraviolet time-resolved photoelectron spectroscopy

A. von Conta[1], A. Tehlar[1], A. Schletter[1], Y. Arasaki[2], K. Takatsuka[2] and H. J. Wörner[1]

[1]Laboratory for Physical Chemistry, ETH Zurich, Wolfgang-Pauli-Strasse 10, CH-8093 Zurich, Switzerland

[2]Fukui Institute for Fundamental Chemistry, Kyoto University, Sakyo-ku, Kyoto 606-8103, Japan

June 2, 2018

[Supplementary Information]

Supplementary Note 1: Wavepacket dynamics

The wave packet calculations were performed with the method presented in [2]. Here, we present the results of a simulation with similar parameters compared to the experiment: the pump pulse is centered at 400 nm with a gaussian envelope of the intensity of 40 fs FWHM and a peak intensity of $2 \cdot 10^{11}$ W/cm^2 which is centered at $t = 0$ fs.

Supplementary Figure 1a illustrates the populations of the electronic states in both the diabatic and the adiabatic frame. Due to the limited size of the grid, absorbing boundaries are introduced. In this calculation the absorption barriers are located at $r_{1/2} = 3.0125$ Å, leading to a loss of the total population if the WP accesses this part of the nuclear configuration space (see Fig. 1b and c). To illustrate the evolution of the excited WP, all parts of the WP overlapping with the GS wave function are removed by an appropriate projection. An accessible picture of the time-dependent motion of the WP is obtained by integrating the resulting wave function over two coordinates to obtain a 2-D density map using

$$\rho_{r,i} = \int \left|\chi_i^{\text{proj}}(r_1, r_2, \Theta)\right|^2 \mathrm{d}r_2 \mathrm{d}\Theta \tag{1}$$

$$\rho_{\Theta,i} = \int \left|\chi_i^{\text{proj}}(r_1, r_2, \Theta)\right|^2 \mathrm{d}r_1 \mathrm{d}r_2, \tag{2}$$

where χ_i^{proj} is the vibrational wave function without ground state contributions of the state i. Note that r_1 and r_2 are not distinguishable. The two quantities are shown in fig. 1b and fig. 1c, respectively.

The marked timings in Supplementary Figure 5 of the main text are denoted by the vertical lines in Supplementary Figure 1. The photoelectron band 2 (c.f. Supplementary Figure 5i) between 8.8 and 10.7 eV reaches an intensity plateau at 78 fs (red vertical lines), which coincides with the WP reaching large bond lengths larger than 2.25 Å(panel c). This agrees well with the behavior of the vertical I_p (cf. fig. 5d) predicting the generation of signal in this band at large r.

The photoelectron band 3 (c.f. Supplementary Figure 5j of the main text) between 11.7 and 12.6 eV has a local maximum at 36 fs (black vertical lines), which coincides with a fast change in the adiabatic population (panel a). At this delay a large part of the WP passes through the conical intersection and reaches the adiabatic ground state.

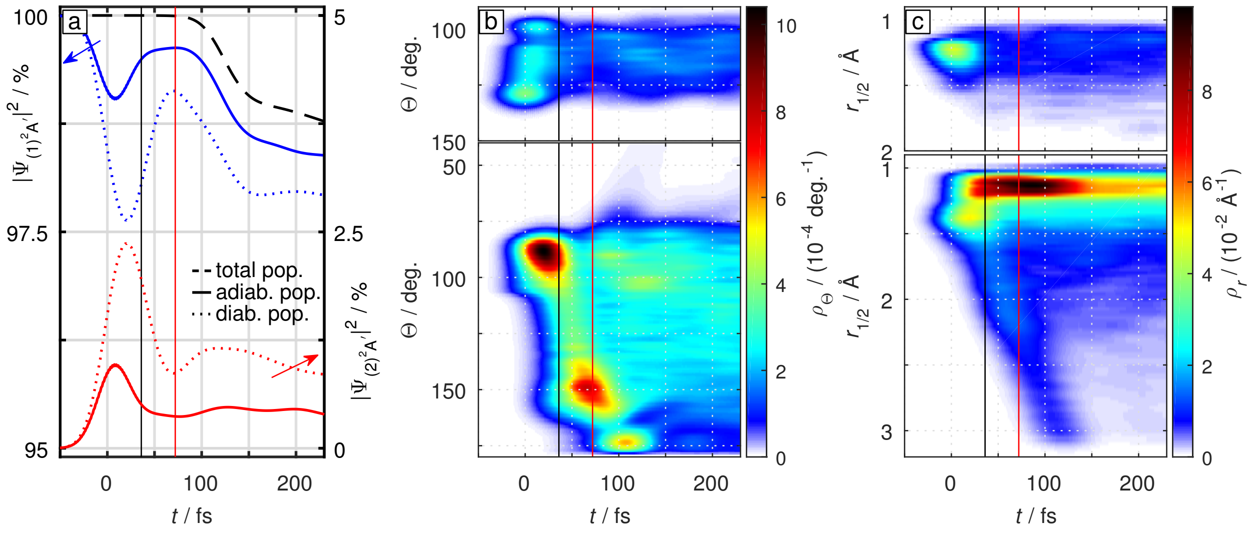

Supplementary Figure 1: Time-dependent wave-packet propagation with a pump pulse comparable to the experiment. a) Time-dependent populations in the $(1)^2\mathrm{A}'$ and $(2)^2\mathrm{A}'$ states. The populations of the energetically lower states are denoted in blue with the scale on the left, the populations of the energetically higher states are denoted in red with the scale on the right. Diabatic populations have dotted lines, adiabatic states have solid lines, and the total population is depicted in black (left scale). b) Time dependent density along the bonding angle Θ (see equation 2) of the adiabatic $(2)^2\mathrm{A}'$ state in the upper panel and of the adiabatic $(1)^2\mathrm{A}'$ state in the lower panel. c) Time dependent density along $r_{1/2}$. Because r_1 and r_2 are not distinguishable, they both contribute to the density - i.e. large $r_{1/2}$ always imply an equal density at $r_{2/1}$ around the equilibrium position of r_{NO}. The highlighted timings (black at 36 fs and red at 72 fs) are associated with characteristic structures in the photoelectron bands (see text).

Supplementary Note 2: Experimental techniques

Experimental layout. A Ti:Saph amplifier system delivers 30 fs laser pulses at 5 kHz, centered at 800 nm, and with a pulse energy of 2 mJ. The incident beam is split into two interferometric arms. 1 mJ is used to generate high-order harmonics in a semi-infinite cell [15]. After this a single harmonic order is isolated by a time-preserving monochromator [10], delivering XUV pulses of about 35 fs with a spectral bandwidth on the order of 300 meV. The UV pulses are generated in the second interferometric arm by second-harmonic generation using a 100 μm thick BBO crystal with a cut angle of 29.2°. 1 mJ of input energy yields 250 μJ UV pulses (25% conversion efficiency) with a duration of 44 fs. The diameter of the UV beam is reduced by an iris to $\sim$ 2 mm, resulting in pulse energies of 3-5μJ. The XUV and UV beam are then recombined in an angle-integrating magnetic-bottle time-of-flight photoelectron spectrometer (MBES) [7], whereby the UV beam is focused by a mirror with a focal length of 1 m. The diameter of the XUV focus is estimated to amount to 100-150 μm. The measured XUV-UV cross correlation amounts to about 56 fs. A beam-path overview is given in Supplementary Figure 2.

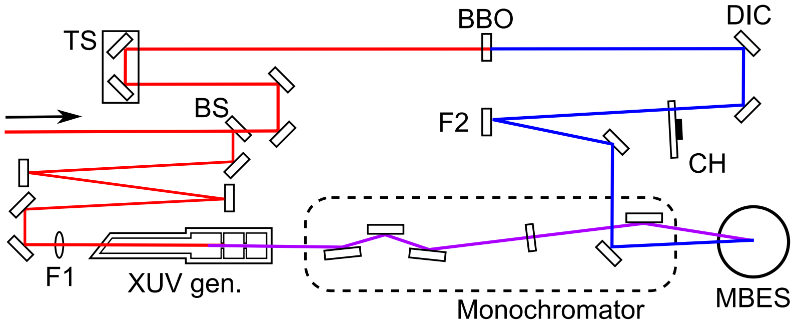

Supplementary Figure 2: The beamsplitter BS is used to separate the incoming IR radiation in two interferometric arms. The reflection off the BS is focused with the lens F1 into the semi-infinite gas cell for XUV generation and consecutive monochromatization. The transmitted part is frequency doubled using a BBO crystal whereafter the remaining IR is separated using dichroic mirrors (DIC). Before focusing the UV via F2, a chopper CH is employed to block every second pulse thereby allowing for single-shot-referenced detection. The two light pulses are then combined in the MBES. The optical path length of the UV pulses can be changed using a motorized translation stage TS.

For each dataset the excitation spectrum was recorded as well as a cross correlation in argon. The excitation spectrum used for the fs-dynamics dataset shown in the main body of the text is given in Supplementary Figure 3a. By tilting the BBO crystal, the spectrum could be shaped, moving its center of gravity by $\pm$5 nm around 400 nm. The shown spectrum is tuned to about 399 nm and slightly asymmetric due to the tilt dependent phase-matching in the BBO. Suplementary Figure 3b shows the cross correlation recorded for the same data set. Typical values for the FWHM of the cross correlation are around 55 fs depending on day-to-day alignment and compressor settings.

The MBES was calibrated before each set of measurements using photoelectron spectra from argon, obtained with XUV pulses corresponding to different harmonic orders. A continuous-flow needle-type leak valve was used to supply the gas into the MBES. It consists out of a 40 mm long, electrically-grounded stainless steel tube with an inner diameter of 127 μm which is positioned at a distance of 1 mm relative to the interaction region. The backing pressure of the leak valve was actively stabilized by a membrane gauge and

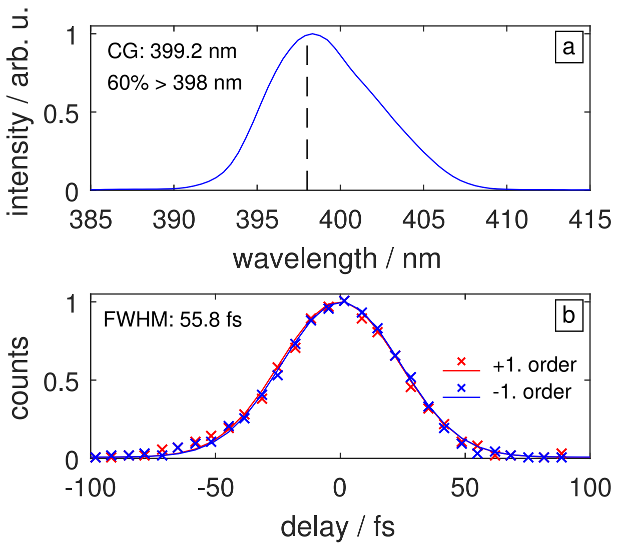

Supplementary Figure 3: Excitation spectrum and argon cross correlation measured for the fs-daynamics dataset presented in the main body of the text. a) UV excitation spectrum. The spectrum is centered around 399 nm and about 60% of the spectral intensity is above the dissociation threshold. b) cross correlation measured prior to the NO_2 data set in argon. A gaussian is fit (solid line) is performend on the experimental data (crosses) for both first order side bands. The average FWHM is 55.8 fs which indicates a UV pulse duration around 44 fs.

an actuated precision-needle valve.

In order to prevent cluster formation in NO_2, the leak valve is heated to 90°C with a temperature-stabilized heating foil. The equilibrium constant for the reaction $NO_2 \leftrightarrow N_2O_4$ is $K_\mathrm{p} = p^2_{\mathrm{NO_2}}/p_{\mathrm{N_2O_4}} \approx 7$ atm. at 359.6 K (86.6°C) and a pressure of 40 mbar [1]. This yields a fraction of N_2O_4 of 11% assuming a thermally-equilibrated sample.

Methodology of the Data Processing. The data acquisition is set up around a 2-channel high-speed 8 bit analog-to-digital converter card with an internal ringbuffer. The MCP signal is acquired for each shot and stored in the ringbuffer which can be read out simultaneously without loss of trigger events. The read out data is then transformed into a binary representation by appropriate thresholding, removing spurious RF noise. The second channel is used as qualifying input to determine the chopper status, i.e. whether a trigger event contained only the XUV pulse or both pulses. The total electron-count-rate per shot, was set sufficiently low to avoid double counts within the time window defined by the single event response of the MCP, including the RF ringing. This corresponds to about 50 registered electron counts per shot.

For each delay step, 3×10^5 trigger-events were acquired, corresponding to 1.5×10^5 UV + XUV events. The data sets, shown in the main body of the text, all consist of 20 independent delay scans which were averaged, corresponding to 3×10^6 trigger events contributing to the average ($\approx 1.5 \times 10^8$ electron counts), corresponding to an integration time of 10 minutes per delay step. For the shown data sets 110 delay steps were recorded per scan, with a delay step of 6.33 fs and 33.3 fs for the short- and long-term dynamics respectively.

The time-of-flight spectra are then converted to photo-electron kinetic energy by a suit-

able calibration function where after the data was rebinned on an equidistant grid with a spacing of 50 meV. During the acquisition time of 36 hours the XUV pulses drifted in their flux (by 50 %) and in their corresponding photon energy (by 150 meV). The average total number of XUV only counts per delay-step per scan was calculated and for each delay step both the XUV and the XUV + UV spectrum were normalized accordingly to account for a time-dependent drift of the XUV flux. The time-dependent shift of the XUV photon energy was partially compensated by calculating individual binding energy axes for each scan and interpolating on a common grid before averaging. The data sets are not compensated for a possible drift in UV pulse energy ($\approx$ 30 % over the duration of the scan).

The resulting delay averaged XUV and XUV + UV spectra are shown in Supplementary Figure 4 on a kinetic energy axis. For this data set the direct ionization by the pump pulse accounts for about 10 % of the collected electrons in the XUV + UV spectra. The direct ionization dominates the difference spectrum at low kinetic energies up to about 3 eV.

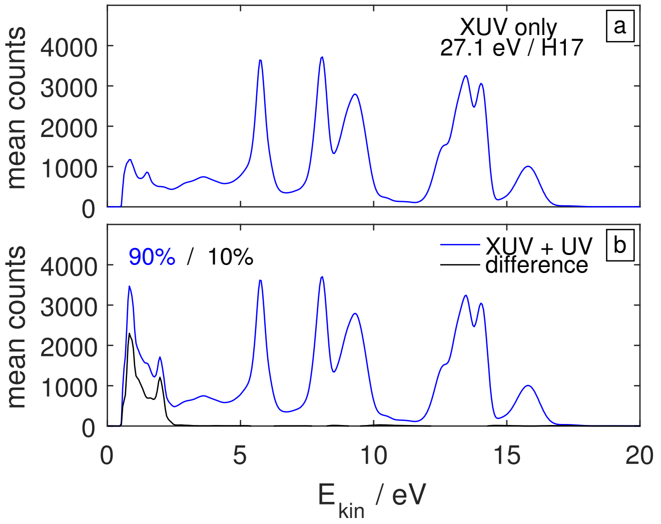

Supplementary Figure 4: Photoelectron spectra obtained in NO_2. a) XUV spectrum. b) Spectrum obtained in the presence of UV and XUV. The UV ionization accounts for 90 % of the ionization events in the XUV + UV case.

The resulting time-dependent average XUV and XUV + UV spectra, calculated from the independent scans, are shown in Supplementary Figure 5a-b in terms of binding energy, excluding the low kinetic energy part at high binding energies. The resulting difference spectrum is shown in Supplementary Figure 5c. The obtained differences are on the order of 50 counts given a peak count in the reference of 3700. Due to the small signal size it was decided to apply oversampling in the time-delay dimension, i.e. the delay step size of 6.3 fs is significantly smaller than the duration of the cross correlation (56 fs). A moving average can therefore be applied for displaying purposes, without losing significant time resolution. If the data sets are shown as 2D map, they have been convolved with Gaussians (see Supplementary Figure 5d).

Methodology of the Error Analysis. As can be seen from Supplementary Figure 5, the error analysis in terms of statistical fluctuations of individual data points is essential. In order to quantify this the standard deviation of the average was calculated for each

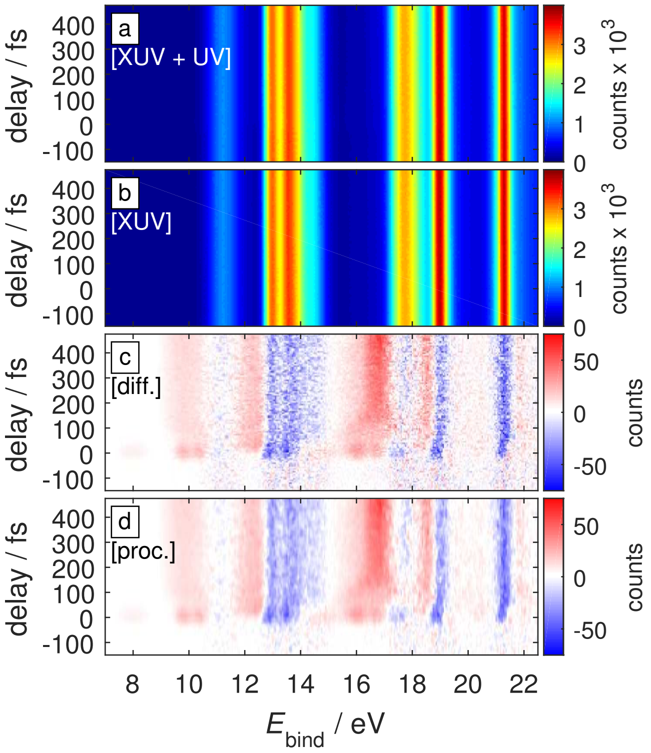

Supplementary Figure 5: Raw and processed data after energy calibration, rebinning, photon-energy drift compensation and averaging. a) Averaged raw XUV + UV spectrum. b) Averaged raw XUV-only spectrum. c) Difference spectrum calculated from a) and b). d) Difference spectrum after application of the moving average by convolution with a Gaussian in both time (FWHM: 18 fs) and energy (FWHM: 100 meV).

individual energy bin in the mean difference spectrum. This information can then be used to calculate the standard deviation of integrated bands by Gaussian error propagation. Suplementary Figure 6a-c, shows the standard deviation of the average for the XUV and the XUV + UV and calculated standard deviation of the difference. As can be seen in Supplementary Figure 6b-c the standard deviation of the average can be as high as 10 % of the peak count value in the XUV only spectrum.

The most significant contribution to the statistical variation of the data stems from a time-dependent drift of the XUV photon energy or XUV bandwidth during a scan, which can be seen close to sharp edges of the XUV only spectrum. The band edge at 12.8 eV for example causes a peak in the standard deviation with a FWHM of $\approx$ 250 meV, giving an estimate of the magnitude of the fluctuation. This implies that the statistical fluctuations of integration bands, in the vicinity of a sharp feature, are orders of magnitude larger than the overall small signals, making the data statistically insignificant without further processing.

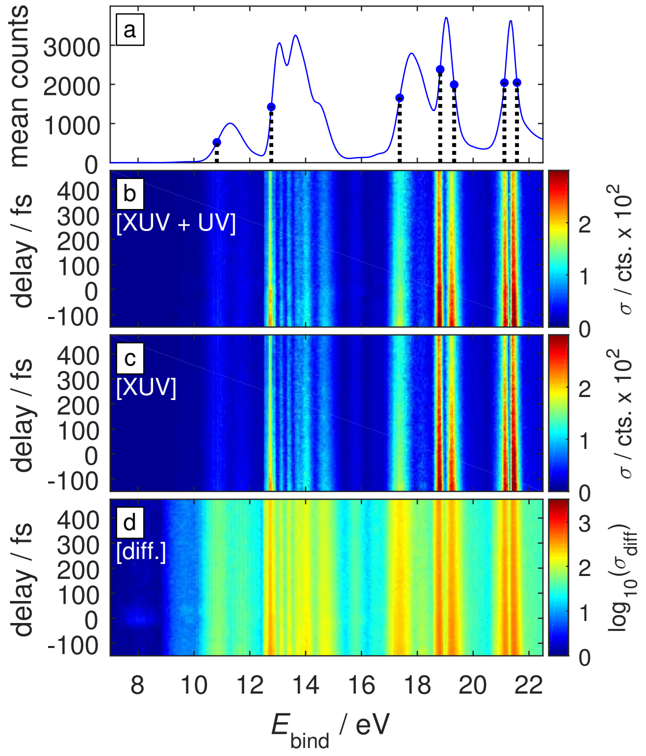

Supplementary Figure 6: Standard deviation of the average calculated for each individual time and energy bin for the average XUV + UV spectrum (b), the average XUV only spectrum (c) and the resulting difference (d). The mean XUV only spectrum is shown in (a). The dashed lines highlight the steepest edges which coincide with the regions of high statistical fluctuations.

Supplementary Note 3: LAPE calculations

The strong-field approximation (SFA), also known as Keldysh-Faisal-Reiss theory [12], has been proven to be useful for such a broad range of problems across the spectrum of light-matter interaction, that it has become textbook knowledge [6, 14]. This framework can be used to calculate photoelectron spectra in the presence of the laser assisted photoelectric effect (LAPE).

LAPE is an effect in which an ionized electron interacts with an assisting electric field to exchange one or more photons in the presence of the remaining ion, thereby changing its kinetic energy and angular distribution. LAPE can be conceptualized, in the case of an initial XUV single-photon ionization, as

$$\mathrm{A}(i) + \hbar\omega_{XUV} \rightarrow \left[\mathrm{e}^-(\vec{p}) + n\hbar\omega\right] + \mathrm{A}^+(f) \rightarrow \mathrm{e}^-(\vec{p'}) + \mathrm{A}^+(f), \tag{3}$$

where $\mathrm{A}(i)$ denotes an atom in its initial state, $\mathrm{A}^+(f)$ denotes the cation in its final state after ionization with the XUV photon $\hbar\omega_{XUV}$, $n\hbar\omega$ implies the exchange of n photons of the assisting field with energy $\hbar\omega$, and $\mathrm{e}^-(\vec{p})$ is an electron with kinematic momentum $\vec{p}$. A simple expression can be derived, using a binding-energy dependent experimental XUV spectrum $\mathcal{S}_{\mathrm{XUV}}(E_{\mathrm{bind}})$ as input, to calculate the time-dependent effective probability to obtain an electron with momentum $\vec{p'}$, allowing for several, however non-interacting, final states f. The evaluated expression, neglecting the angular distribution of the ionization step, is

$$\begin{aligned} P(|\vec{p}|, \tau) \;\propto\; & \int_{\mathcal{S}} \mathcal{S}_{\mathrm{XUV}}(E_{\mathrm{bind}}) \Big| \int_{-\infty}^{\infty} \mathcal{E}_{\mathrm{XUV}}(t' - \tau) \exp\left(\frac{i}{\hbar}\frac{|\vec{p}|^2}{2m_e} t'\right) \\ & \times \exp\left(-\frac{i}{\hbar}\frac{e}{m_e}\int_{t'}^{\infty} |\vec{p}|\,|\vec{A}(t'')|\mathrm{d}t''\right) \exp\left(-\frac{i}{\hbar}\frac{e^2}{2m_e}\int_{t'}^{\infty} |\vec{A}(t'')|^2 \mathrm{d}t''\right) \\ & \times \exp\left(\frac{i}{\hbar} E_{\mathrm{bind}}\, t'\right) \mathrm{d}t' \Big|^2 \mathrm{d}E_{\mathrm{bind}}, \end{aligned} \tag{4}$$

where $\mathcal{E}_{\mathrm{XUV}}(t' - \tau)$ represents the electric field of the XUV shifted by a time delay τ relative to the assisting field, m_e is the mass of the electron, e is the electron charge, $\vec{A}(t)$ is the vector potential of the assisting field, and E_{bind} is the binding energy. In this expression, which can be evaluated on a numerical grid, each spectral contribution in $\mathcal{S}(E_{\mathrm{bind}})$ is treated as a transition to a non-interacting final state. The results can be compared to experimental spectra by normalizing the spectrum accordingly, e.g.

$$\frac{\mathcal{S}_{\mathrm{exp}}(E_{\mathrm{bind}}, \tau)}{\max(\mathcal{S}_{\mathrm{XUV}}(E_{\mathrm{bind}}))} = \frac{P(|\vec{p}(E_{\mathrm{bind}})|, \tau)}{\max(P_{\mathrm{XUV}}(|\vec{p}(E_{\mathrm{bind}})|))}, \tag{5}$$

where $P_{\mathrm{XUV}}(|\vec{p}(E_{\mathrm{bind}})|)$ is calculated in the absence of the assisting field.

Supplementary Note 4: Depletion correction

An essential technique in unravelling the dynamics of the UV-induced excited-state fraction is the possibility to compensate the experimental normalized-difference spectra for depletion effects. The idea is that the contribution of the unperturbed ground state photoelectron spectrum $\mathcal{S}_{\mathrm{GS}}(E_{\mathrm{bind}})$ is directly proportional to the population $|c_{\mathrm{GS}}(t)|^2$ of the lowest vibrational level of the electronic ground state (GS):

$$\mathcal{S}_{\mathrm{GS}}(E_{\mathrm{bind}}, t) \propto |c_{\mathrm{GS}}(t)|^2. \tag{6}$$

Provided there is only a single electronic final state and provided the depletion and induced dynamics in the electronic ground state are small, the population transfer will follow the real-valued field envelope $\mathcal{A}(t)$ of the interacting pulse, assuming a simple two-level scheme. This implies that

$$\mathcal{S}_{\mathrm{GS}}(E_{\mathrm{bind}}, t) \propto 1 - \alpha \left(\int_{-\infty}^{t} \mathcal{A}(t')\mathrm{d}t' \right)^2, \tag{7}$$

where α is a parameter depending on the $\vec{R}$ dependent transition dipole moment and the detuning. This leads to the following heuristic function for the time-dependent photoelectron spectrum

$$\mathcal{S}_{\mathrm{GS}}(E_{\mathrm{bind}}, t) = \mathcal{S}_{\mathrm{XUV}}(E_{\mathrm{bind}}) \left(1 - \alpha \left(\int_{-\infty}^{t} \mathcal{A}(t')\mathrm{d}t' \right)^2 \right), \tag{8}$$

where $S_{\mathrm{XUV}}(E_{\mathrm{bind}})$ denotes the XUV-only spectrum. The resulting correction function is then

$$\Delta^{\mathrm{corr}}(E_{\mathrm{bind}}, t) = \mathcal{S}_{\mathrm{XUV}}(E_{\mathrm{bind}})\, \alpha \left(\int_{-\infty}^{t} \mathcal{A}(t')\mathrm{d}t' \right)^2. \tag{9}$$

We can now introduce the empirical depletion parameter $\beta = \alpha \left(\int_{-\infty}^{\infty} \mathcal{A}(t')\mathrm{d}t' \right)^2$ to obtain

$$\Delta^{\mathrm{corr}}(E_{\mathrm{bind}}, t) = \mathcal{S}_{\mathrm{XUV}}(E_{\mathrm{bind}})\, \beta \left(\frac{\int_{-\infty}^{t} \mathcal{A}(t')\mathrm{d}t'}{\int_{-\infty}^{\infty} \mathcal{A}(t')\mathrm{d}t'} \right)^2. \tag{10}$$

Assuming that the UV pulse has a Gaussian envelope with a FWHM of σ_{t} which can be deduced from experiments, this leaves only β as a free parameter.

In order to extract β from experimental data, suitably located depletion and gain bands in the difference spectrum are required. Ideally, a single free standing depletion feature is available to extract the excitation fraction directly. If this is not the case, regions of the spectrum can be exploited where a depletion sharply intersects with a gain band. β is then varied until a smooth transition is achieved from areas influenced by the depletion to areas free of depletion effects in the corrected difference spectrum . This works because most gain bands turn out to be broad homogeneous structures devoid of sharp edges. Clearly the latter technique has a larger error margin than the first.

Suplementary Figure 8 shows the depletion correction being applied to one of the data sets discussed in the main body of the text. The pulse duration σ_t is set to 44 fs (see Supplementary Figure 3). In order to determine β, $\bar{\Delta}^{\mathrm{corr}}_{\mathrm{norm}}$ is evaluated for time-delays outside the pulse overlap. As a first step, β is gradually increased until there are no unphysical negative contributions left (see the arrow annotation at 21 eV in Supplementary Figure 8d). Then the β is further increased until unphysical edges start appearing (see the arrow annotation at 13 eV in Supplementary Figure 8d). Typically, β yields values showing an *unphysicality-boundary* on the order of $\pm$ 30%. In order to compare the different obtained corrected normalized difference spectra qualitatively, $\bar{\Delta}^{\mathrm{corr}}_{\mathrm{norm}}$ is shown for three different values of β.

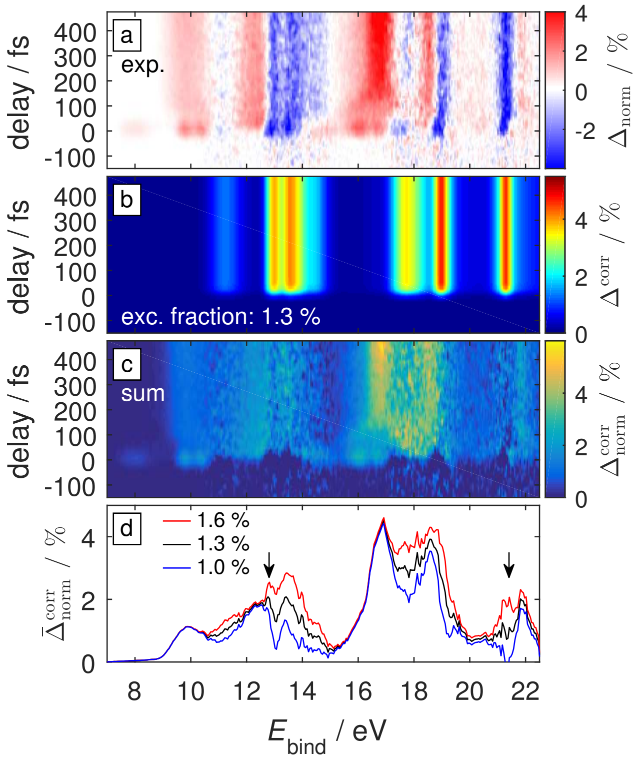

Supplementary Figure 7: Illustration of the depletion correction. a) processed normalized difference spectrum. b) correction function described in the text assuming an excitation fraction of 1.3 %. c) corrected spectrum obtained by summation of a) and b). d) delay averaged spectra calculated for different assumed excitation fractions. The two positions marked by the arrows show unphysical behavior for excitation fractions within ±30% of the assumed value (see text).

We have further studied the sensitivity of the depletion correction to the selected delay interval and found a very low sensitivity. Suplementary Figure S11 shows the depletion corrected spectra $\bar{\Delta}^{\mathrm{corr}}_{\mathrm{norm}}$ obtained by using three different delay intervals for the normalization procedure as indicated in each of the panels. Within the achieved signal-to-noise ratio, no clear dependence on the chosen delay interval can be observed, as expected.

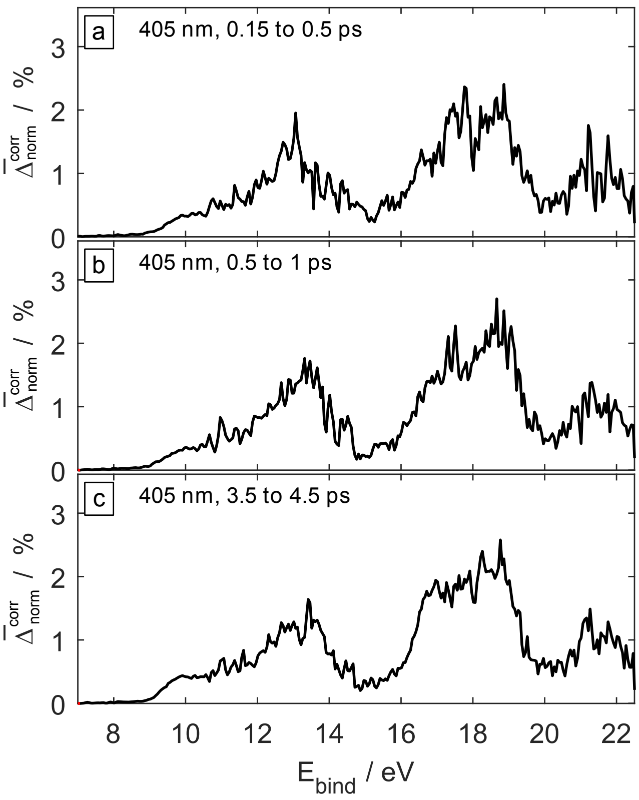

Supplementary Figure 8: Sensitivity of the depletion correction to the chosen delay interval. The selected interval is indicated in each panel. This data set corresponds to the results shown in Fig. 6, panels c and e, i.e. for an excitation spectrum centered at 405 nm.

Supplementary Note 5: Calculation of photoelectron spectra

The photoelectron spectra were calculated within the Born-Oppenheimer approximation and the semiclassical approximation [8] leading to

$$\sigma(E_{\rm bind}) = \frac{\pi e^2 E_{h\nu}}{3\epsilon_0 c} \sum_{i,f} \int \left|\mu_{if}\left(\vec{R}\right)\right|^2 \left|\chi_{\rm i}\left(\vec{R},t\right)\right|^2 \delta\left(E_{\rm bind} - I_{\rm p}^{if}\left(\vec{R}\right)\right) {\rm d}\vec{R}, \tag{11}$$

where $E_{h\nu}$ is the photon energy, μ_{if} is the electronic transition dipole, χ is the nuclear wave-function, and $I_{\rm p}^{if}$ is the ionization potential. A similar approach was presented in [13].

The calculation of the photoelectron spectra contains 16 different channels: from the adiabatic $(1)^2\mathrm{A}'$ and $(2)^2\mathrm{A}'$ states of the neutral molecule to the energetically lowest two states of each, the $^1\mathrm{A}'$, $^1\mathrm{A}''$, $^3\mathrm{A}'$, and $^3\mathrm{A}''$ of the cationic molecule. The magnitude of the transition dipole μ_{if} was approximated by the norm of the Dyson orbital, assuming sudden ionization and a constant density of states in the continuum [4, 9]. This is a measure of the electronic overlap of the cationic molecule with the neutral molecule missing the ionized electron.

Supplementary Figure 9 shows a comparison of experimental spectra with the calculated photoelectron spectrum of the calculated nuclear ground-state wave function. As in the main body of the text, the spectra are normalized to the $^1\mathrm{A}'$ peak of the photoelectron spectrum. The calculated spectrum shows excellent agreement with the XUV photoelectron spectrum shown in this work. The relative peak height of the $^1\mathrm{A}'$ contribution is, however, overestimated. This calculation underlines the feasibility of the proposed method.

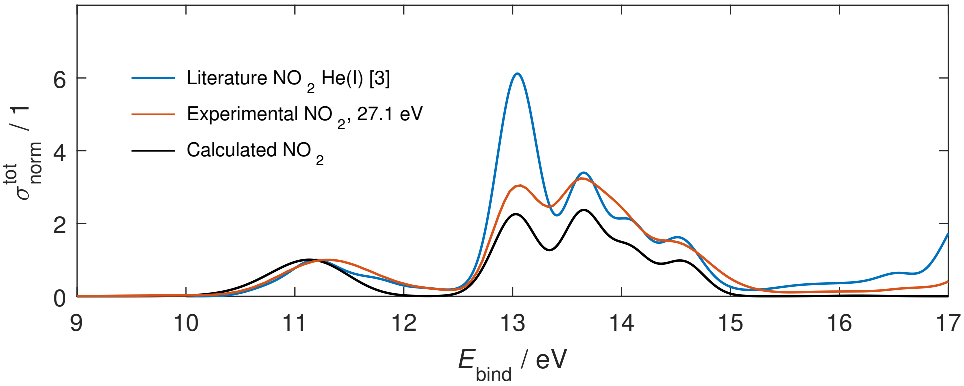

Supplementary Figure 9: Calculated spectrum of the NO_2 ground state (red) compared to an experimental spectrum (green) and a high-resolution spectrum from literature [3] convolved with a gaussian of 0.35 eV FWHM (red).

The norm of the transition amplitude $|\mu_{if}|^2$ can be expressed within the sudden ionization approximation as the norm of the Dyson orbital [5, 11]. This quantity is varying as a function of the nuclear coordinates, as is shown in Supplementary Figure 10. For example, the small electronic overlap of the excited state with the cationic states at the equilibrium

position (indicated by an arrow in the figure), leads to a suppression of the signal directly after the excitation. This property gives XUV-TRPES its unique sensitivity to changes in electronic character of the WP.

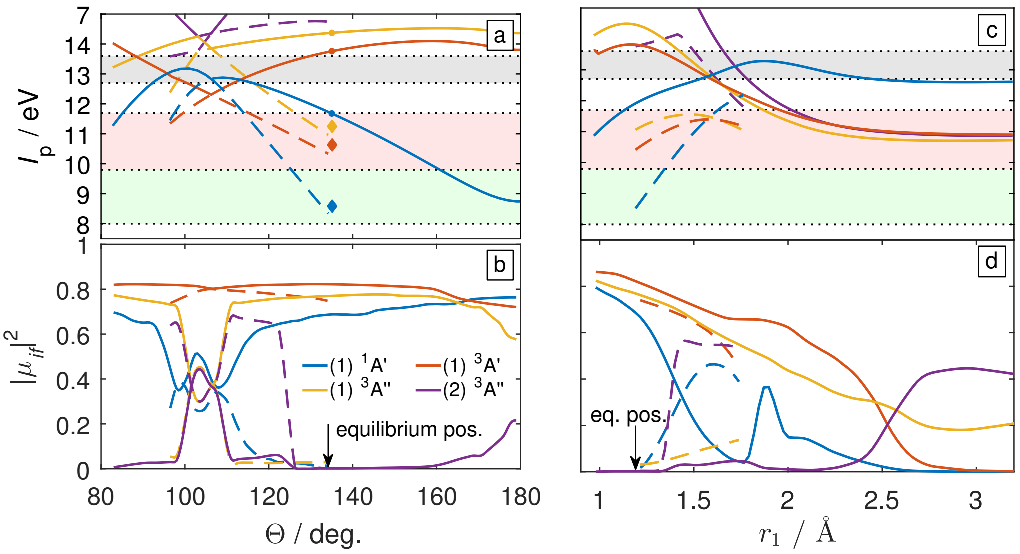

Supplementary Figure 10: Norm of the Dyson orbital as a function of Θ and r_1. a) and c) illustrate the vertical I_{p} of different channels in dependence of the bond angle Θ (panel a) and the bond length r_1 (panel c). The other degrees of freedom are set to the equilibrium value of NO_2. Panel b) and d) show the norm of the Dyson orbital of the main channels in dependence of Θ and r_1.

Equation 11 allows the separate calculation of the different channels. An example of the time dependent contribution of the ionization from the $(1)^2\mathrm{A}'_{\mathrm{A}}$ state to the $(1)^3\mathrm{A}''_{\mathrm{A}}$ state to the photoelectron signal is shown in Supplementary Figure 11. Panel a shows the direct result of the equation 11, convolved with Gaussians according to the approximated resolutions of the experiment (35 fs FWHM for the probe pulse intensity envelope and 0.35 eV FWHM for the energy resolution).

As pointed out in section in the supplementary, the finite extent of the grid employed for the WP calculations requires the implementation of absorbing boundaries. The part of the wave packet, which is absorbed at the boundary of the grid, is approximated with an average spectrum calculated from the average density in the absorbing volume. The contribution of the absorbed population is added in panel b of Supplementary Figure 11. The experimental observable is corresponding to the summation of the individual channels including their boundary contributions (see Supplementary Figure 11c).

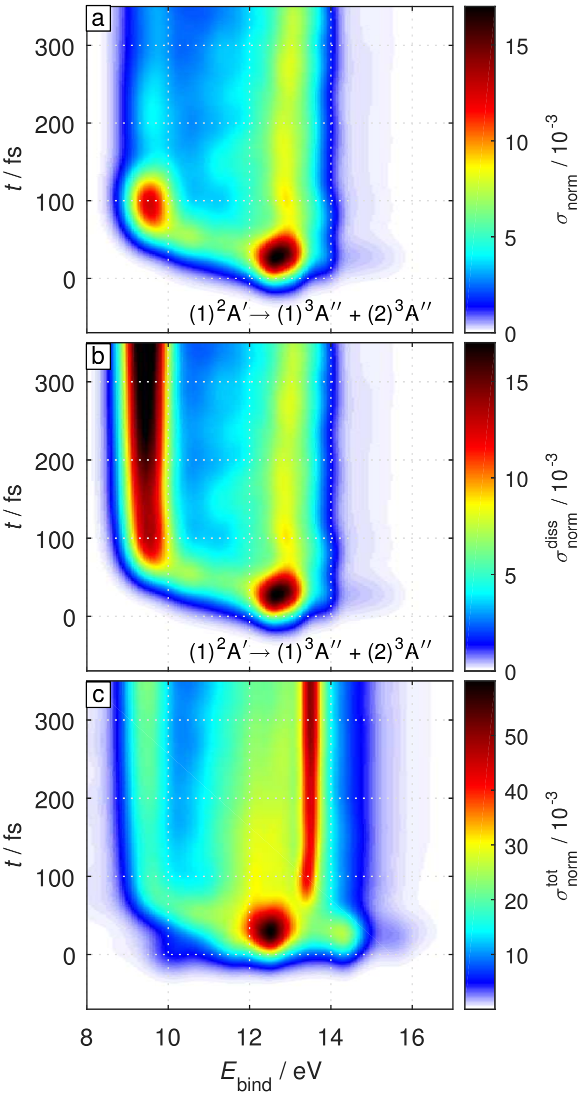

Supplementary Figure 11: Calculated time-dependent photoelectron spectra of NO_2 without ground state contributions. a) time-dependent photoelectron spectra due to the transition from $(1)^2\mathrm{A}'$ to the two energetically lowest lying $^3\mathrm{A}''$ states without contribution of the absorbed part of the wave function (see text). b) same as a) but with the additional contribution of the absorbed wave function. c) total calculated time-dependent photoelectron spectra. The cross-sections are normalized with respect to the maximal intensity of the $(1)^2A_1$ to $(1)^1A_1$ band at 11.1 eV of the static spectrum.

Supplementary References

[1] Antonella Amoruso, Luca Crescentini, Giorgio Fiocco, and Massimiliano Volpe. New measurements of the NO_2 absorption cross section in the 440- to 460-nm region and estimates of the NO_2-N_2O_4 equilibrium constant. *J. Geophys. Res.*, 98(D9):16857–16863, 1993.

[2] Yasuki Arasaki and Kazuo Takatsuka. Quantum wavepacket dynamics for time-resolved photoelectron spectroscopy of the NO_2 conical intersection. *Chemical Physics*, 338(2):175–185, September 2007.

[3] P. Baltzer, L. Karlsson, B. Wannberg, D. M. P. Holland, M. A. MacDonald, M. A. Hayes, and J. H. D. Eland. An experimental study of the valence shell photoelectron spectrum of the NO_2 molecule. *Chemical Physics*, 237(3):451–470, October 1998.

[4] Gilbert Grell, Sergey I. Bokarev, Bernd Winter, Robert Seidel, Emad F. Aziz, Saadullah G. Aziz, and Oliver Kühn. Multi-reference approach to the calculation of photoelectron spectra including spin-orbit coupling. *The Journal of Chemical Physics*, 143(7):074104, August 2015.

[5] Gilbert Grell, Sergey I. Bokarev, Bernd Winter, Robert Seidel, Emad F. Aziz, Saadullah G. Aziz, and Oliver Kühn. Erratum: "Multi-reference approach to the calculation of photoelectron spectra including spin-orbit coupling" [J. Chem. Phys. 143, 074104 (2015)]. *The Journal of Chemical Physics*, 145(8):089901, aug 2016.

[6] C. J. Joachain, N. J. Kylstra, and R. M. Potvliege. *Atoms in Intense Laser Fields*. Cambridge University Press, Cambridge, 2011.

[7] P. Kruit and F.H. Read. Magnetic field paralleliser for 2π electron-spectrometer and electron-image magnifier. *Journal of Physics E: Scientific Instruments*, 16(4):313–324, 1983.

[8] Melvin Lax. The franck-condon principle and its application to crystals. *The Journal of Chemical Physics*, 20(11):1752–1760, nov 1952.

[9] R. Manne and T. Åberg. Koopmans' theorem for inner-shell ionization. *Chemical Physics Letters*, 7(2):282–284, October 1970.

[10] Luca Poletto and Paolo Villoresi. Time-delay compensated monochromator in the off-plane mount for extreme-ultraviolet ultrashort pulses. *Appl. Opt.*, 45(34):8577–8585, Dec 2006.

[11] Aurora Ponzi, Celestino Angeli, Renzo Cimiraglia, Sonia Coriani, and Piero Decleva. Dynamical photoionization observables of the CS molecule: The role of electron correlation. *The Journal of Chemical Physics*, 140(20):204304, may 2014.

[12] Howard R. Reiss. Effect of an intense electromagnetic field on a weakly bound system. *Phys. Rev. A*, 22(5):1786–1813, November 1980.

[13] Fabio Della Sala, Roger Rousseau, Andreas Görling, and Dominik Marx. Quantum and thermal fluctuation effects on the photoabsorption spectra of clusters. *Physical Review Letters*, 92(18):183401, may 2004.

[14] Olga Smirnova and Misha Ivanov. *Multielectron High Harmonic Generation: Simple Man on a Complex Plane*, pages 201–256. Wiley-VCH Verlag GmbH & Co. KGaA, 2014.

[15] Daniel S. Steingrube, Tobias Vockerodt, Emilia Schulz, Uwe Morgner, and Milutin Kovačev. Phase matching of high-order harmonics in a semi-infinite gas cell. *Phys. Rev. A*, 80:043819, Oct 2009.
